# The Action of Rattlesnake Venom upon the Bactericidal Power of the Blood-serum1From the proceedings of the Washington meeting of military surgeons.

**Published:** 1895-03

**Authors:** Charles B. Ewing

**Affiliations:** Medical Department, United States Army


### THE ACTION OF RATTLESNAKE VENOM UPON THE BACTERICIDAL POWER OF THE BLOOD-SERUM.[1]

BY CHARLES B. EWING, M.D.,
MEDICAL DEPARTMENT, UNITED STATES ARMY.

At Professor Welch's suggestion we conducted a series of experiments in the pathological laboratory of the Johns Hopkins University during the spring of 1893, having for their purpose the determination of the action of rattlesnake venom upon the bactericidal power of the blood.

Dr. Welch's attention was directed to the investigation of this subject by Dr. Weir Mitchell. Drs. Mitchell and Reichert demonstrated that the poisonous properties of rattlesnake venom depend upon the presence of proteid substances. These investigators were the first to demonstrate the existence of the so-called toxic albumins.

In their monograph on the subject, as well as from previous observations, it was apparent that the animals killed by rattlesnake venom decomposed with great rapidity—indeed, with

[1] From the proceedings of the Washington meeting of military surgeons.

such rapidity that Dr. Formad, who contributed an appendix upon the pathological anatomy of animals dead of rattlesnake venom, believed that there was evidence of the spontaneous generation of bacteria.

Dr. Formad thought it impossible for the bacteria to make their way into the circulation and multiply so quickly. This seemed so improbable to Professor Welch that he suggested to Dr. Weir Mitchell the importance of having this point worked up. Dr. Mitchell kindly gave us a certain amount of poison. We were, however, fortunate enough to obtain a live rattlesnake of the diamond species, known as the *Crotalus adamanteus*, from which fresh venom was obtained and used in preference to the dried poison supplied us.

We had in view, in the first place, to test the question as to whether the blood of animals killed by rattlesnake poison had lost any of its germicidal power.

We used Dr. Weir Mitchell's noose for securing the snake, and with a sterilized saucer thrust into the mouth of the animal we collected the venom. In this way we had no difficulty in getting from 0.5 to 1 c.c. of nearly clear, slightly straw-colored fluid, quite sufficient for our purposes. This was diluted with an equal quantity of sterilized physiological salt solution, and 0.25 to 0.5 c.c. of this mixture was inoculated subcutaneously, under antiseptic precautions, the dose varying according to the time we wished the animal to live. This was usually from one-half to one hour and a half, but in one or two cases three hours after the injection.

The injections were in all cases given subcutaneously, sometimes beneath the skin of the abdomen and at other times in the groin. The lesions differed somewhat, according to the site of injection. When made beneath the skin of the abdomen there were most extensive peritoneal hemorrhages, whereas, when the injections were made in the thigh these lesions were less extensive, although not absent; here the most striking effects noted, beside the deeply discolored integument surrounding the point of injection, were the underlying hemorrhages into the muscles.

The important lesions were these: most extensive hemorrhage, with disintegration of tissue and actual necrosis of tissue, for a wide distance around the point of inoculation, with ecchymosis in other parts of the body, particularly in the serous membranes. Another point which served our purpose admirably was that the blood does not coagulate after death, or coagulates only feebly and after a long interval. . We therefore had no difficulty in collecting a sufficient amount of the fluid blood, and we did so by withdrawing the blood in less than a minute after the animal breathed its last. We exposed the heart at once, and with sterilized instruments made an incision in the right auricle, and with a sterilized pipette we aspirated from the right heart; and then passing the pipette down into the abdominal vena cava, we secured sometimes as much as seven or eight cubic centimetres of blood. This blood was collected in a sterilized test-tube and put in the refrigerator. After twenty-four hours the red blood-corpuscles had settled; sometimes a small, soft, dark coagulum had formed and there was a layer of clear serum.

We pipetted off after twenty-four hours the clear serum, usually collecting 0.5 to 1.5 c.c. The organisms which were used to test the germicidal power of the serum were the bacillus coli communis and the bacillus anthracis. As a rule, we made a control experiment with normal serum; thus we killed a healthy rabbit and withdrew the serum in the same way, collecting the same quantity, and inoculated at the same time a parallel control, tubes with the bacteria to be tested. The cultures were twenty-four to forty-eight hour cultures. Our bacillus anthracis was obtained in suspension from the spleen of an animal recently dead of anthrax. We wished to obtain the anthrax bacillus free from spores, and thought it better to take it fresh from the animal. Similar results were obtained also with twenty-four-hour anthrax cultures grown at room temperature. We made a suspension in salt solution of the organisms, and inoculated a known quantity, one to two platinum loops of the suspension, into the serum tube. Then we made roll cultures.

The following tables, drawn up by Professor Welch, represent only a certain number of our experiments, and are much abbreviated for the sake of clearness:

BACILLUS ANTHRACIS.

*Normal Serum.*

| No. | No. bacilli inoculated. | After 24 hours. | After one week. |
|---|---|---|---|
| 1 A | 485 | 0 | 0 |
| 1 B | 8512 | 0 | 0 |
| 1 C | 210 | 0 | 0 |
| 2 A | 5292 | 0 | 0 |
| 2 B | 1628 | 0 | 0 |

*Venom Serum.*

| No. | No. bacilli inoculated. | After 19 hours. | After 24 hours. |
|---|---|---|---|
| 1 A | 8512 | Countless. | |
| 1 B | 25 | | 3233 |
| 2 | 68 | | 250,000 |
| 3 | 3292 | Countless. | |
| 4 | 3292 | " | |
| 5 | 3292 | " | |

BACILLUS COLI COMMUNIS.

*Normal Serum.*

| No. | No. bacilli inoculated. | Imme-diately. | After 24 hours. | After 48 hours. | After one week. |
|---|---|---|---|---|---|
| 1 A | 250,000 | 256 | 157 | Countless. | Countless. |
| 1 B | 175,000 | 188 | 50 | " | " |
| 2 A | 29,161 | 53 | 0 | 0 | 0 |
| 2 B | 3150 | 18 | 0 | 0 | 0 |
| 3 | 9990 | 13 | 38 | 625 | Countless. |
| 4 | 84,947 | 238 | 0 | 0 | 0 |

*Venom Serum.*

| No. | No. bacilli inoculated. | Imme-diately. | After 22 hours. |
|---|---|---|---|
| 1 | 800,000 | 1077 | Countless. |
| 2 | 3150 | 6 | 26,000 |
| 3 | 16,560 | 32 | Countless. |
| 4 | 9990 | 35 | " |
| 5 | 84,947 | 284 | " |
| 6 | 84,947 | 231 | " |
| 7 | 84,947 | 182 | " |

These experiments were uniform in result and point to one conclusion, that the blood of rabbits killed in one-half to three hours after subcutaneous inoculation with rattlesnake venom has lost its germicidal power. This is of considerable interest, for it is an indication of a very profound alteration in the blood. This germicidal power of the blood is one of very great significance, on which many of the modern theories of immunity depend.

It is of special interest to ascertain under what conditions the germicidal properties of the normal blood-serum are at their highest, and in what way these properties affect the composition of the blood.

The principal workers in this field have been Von Fodor, Nuttall, Wasserman, Kitasato, Buchner, Ogata, Hankin, and others. Von Fodor's work was intended to show that arterial has a more destructive action on bacteria than venous blood, and also that fresh blood has a more destructive action than that which has been standing for some time. It was also found that the germicidal power of the blood was weakened in an atmosphere of oxygen or carbonic acid gas, but the removal of gases from the blood had no appreciable effect.

Temperature affected very materially the bactericidal power of the blood, which increased with the rise of temperature from 38° to 40° C., and then gradually diminished with the fall.

Von Fodor is of the opinion that the individual predisposition of an animal to an infectious disease stands in close relationship with the germicidal power of the blood.

A second series related to the influence of drugs on the power of blood to destroy germs. From experiments the deduction was made that the bactericidal power of the organism was raised by any drug which increased the alkalinity of the blood. The third series verified the conclusions regarding the alkalization of the blood. Of eight rabbits inoculated with anthrax all died, whilst of nineteen which had been previously injected with a solution of soda only three died. A majority of the sixteen remaining were perfectly free from disease, only a few being fatally affected.

Up to this time, however, the doctrine of phagocytosis, as advanced by Metchnikoff, held sway, when Nuttall struck the first severe blow to this theory. He, in his most excellent inaugural dissertation at Göttengen, in 1890, showed that the destruction of virulent bacteria in the blood of animals by the leucocytes was not at all essential, but that the serum of blood free from all cellular elements possessed this power to a degree equal to the blood in its entirety.

Buchner, Lubarsch, Nissen, Stern, and Prudden have practically verified these observations. Buchner, particularly in his experiments upon dogs and rabbits, verified the findings of Nuttall, but went even further, and proved that the bactericidal power of the blood of these animals did not *at all depend* upon the cellular elements, but resided in the clear serum which separated from the clot after the blood had stood a while in a cool place. He also demonstrated that the germicidal action of blood and serum was destroyed by exposure for an hour to 55° C., or by heating to 52° C. for six hours, or to 45.6° C. for twenty hours.

Alternate freezing and thawing did not destroy the bactericidal power of the serum, but it was diminished or completely checked by dialysis with distilled water, or by extreme dilution with the same. He preserved the anti-bacterial action of the serum by making an equal dilution with a six-tenths to seven-tenths per cent. of sodium chloride solution, and was led to believe that the activity of the serum was greater alone than when the cellular elements of the blood were present; hence, he concluded that the active element is a living albumin, having as an essential constituent an alkaline base. This albuminoid substance is thought by Hankin to be identical with his "globulin," isolated from the spleen and lymphatic glands.

According to the views of these experimenters, the germicidal power of the blood resides in the serum alone, and phagocytosis is but a secondary process, the leucocytes taking up the bacteria only after they have been rendered inert by the germicidal power of the serum of the blood and certain other fluids of the body. For our purpose, however, it is not necessary to insist upon the humoral as opposed to the phagocytic doctrine of immunity. All that concerns us is the recognition of the bactericidal power of the blood-serum under certain conditions.

The loss of this normal germicidal power helps us to explain the varying rapidity with which post-mortem decomposition sets in. It is well known that persons dead of different diseases decompose with varying degrees of rapidity. We cannot explain this differing rapidity of decomposition simply by variations in temperature, for under the same external conditions one body will be decomposed in a comparatively few hours and another may remain undecomposed for several days. We selected the animals killed with rattlesnake venom because it is well known that they decompose with great rapidity. The bodies of human beings killed by snake venom are also said to decompose with great rapidity.

The results of our experiments furnish a satisfactory explanation of this phenomenon. The blood at the time of death, and even before death, has lost all, or nearly all, power of resisting the invasion and multiplication of certain bacteria, so that the bacteria of putrefaction which are normally present in the intestine develop with astonishing rapidity, and even before the animal is cold produce this wonderful rapid decomposition. Our experiments are also suggestive as regards certain secondary and mixed infections. The toxic proteids of snake venom belong to the same class of poisons as those formed by toxic bacteria, such as the bacillus of tetanus, of diphtheria, etc.

It is easy to suppose that these infectious diseases may cause a diminution of the germicidal power of the blood against secondary invaders, of which common examples are the pyogenic bacteria, present often in our mouths and intestinal canals, and which, in an individual whose resistance is lowered by a loss of the germicidal power of the blood, may grow and multiply. In other words, we can understand better the causation of many of these secondary infections.

